# Dr. Hamilton

**Published:** 1840-01

**Authors:** 


### OBITUARY.

### JAMES HAMILTON, M.D., F.R.S.E., PROFESSOR OF MIDWIFERY IN THE UNIVERSITY OF EDINBURGH.

Died at his house, in Edinburgh, on the 14th Nov., Dr. Hamilton, the distinguished and popular professor of midwifery, in the 72d year of his age. This melancholy event was the consequence of a febrile attack, induced by a violent degree of exertion and consequent fatigue in the course of his attendance on a case of laborious labour. At first, little danger was apprehended; and although, as time advanced, the symptoms of weakness became more alarming, yet so often did he rally, that, till within a day or two of his demise, strong hopes were entertained that his naturally good constitution would carry him through the attack. The event, however, was otherwise; and he terminated his industrious and useful life on the morning of the day above mentioned, having been confined to the house little more than three weeks.

Dr. Hamilton was born in Edinburgh, in April, 1767. His father, who was his predecessor in that chair which he so long and so ably filled, carefully directed and superintended his son's education, with a view to his succeeding him. His classical and literary studies he prosecuted with equal zeal and success, being remarkable even at that early period for the same quickness of perception and soundness of judgment which characterized him in after life.

In the pursuit of medical knowledge he followed the same course, and the result was the same success. For many years, from the age of eighteen, he rose at five, and till ten in the evening devoted to study all the time he could spare from practice and teaching. And even up to the hour when seized with his last illness, he was an early riser, scrupulous and punctual in the division of his time, and exact in all his engagements.

As a practitioner, few individuals have enjoyed a greater share of public confidence and esteem. From his earliest years he made it a chief object to study the phenomena of disease—thus storing up a vast body of facts, from which he afterwards deduced the great leading principles which guided his practice. To a surprising readiness and tact in the discrimination of disease, he added equal skill in the application of the necessary remedies; at the same time securing the confidence of his patients by his kindly manner and soothing address. It was, however, in his own particular department, when at the bedside of the suffering female, that he shone most conspicuous,—indeed we may say unrivalled; whether we consider the patient perseverance and soothing kindness and sympathy which he displayed in cases of lingering or tedious labour, or the boldness and decision with which he acted in cases of difficulty and danger, when once he had determined on the necessity of interference. It was these qualifications, combined with an intimate knowledge of his profession, which raised him so high, as a safe, successful, and favorite practitioner.

Dr. Hamilton's accomplishments as a teacher were of an equally high order, and were called forth at a very early period of life. In 1788, when only 21, he commenced lecturing as assistant to his father; and in 1800, the whole charge was conferred upon himself; thus for upwards of half a century was he engaged in teaching midwifery and the diseases of women and children. To an intimate knowledge of his subject was added an animated, agreeable, and interesting mode of imparting that knowledge to others, qualities which rarely co-exist—at least in an equal degree—in the same individual, and which combined to render him perhaps the most attractive and successful professor of his day.

At an early period in his professional life he was led to adopt certain innovations upon the usual and established practice. These he had been in the habit of inculcating for many years in his class, but did not fully submit to the public till 1836, when he published his "Practical Observations"—a work embodying the result of an experience extending to upwards of fifty years, and comprehending a practice the most extensive, perhaps, that was ever enjoyed by any single practitioner in this department of medicine. Many of his precepts are now generally adopted by the profession, but the importance and value of others are not as yet admitted by our most distinguished practitioners and teachers. A second edition of this work has just issued from the press, and may be considered, under the melancholy circumstances in which it appears, as his parting legacy to the profession; its final revision and enlargement being the latest object which engaged his attention, previously to his fatal illness.

As to the private character of Dr. Hamilton, we have every reason to believe that the delineation of it given in the public prints of his native city is strictly correct. He is there described as a man of liberal and unostentatious charity to the poor and distressed; as one deeply interested in the success of every beneficent and useful institution; of high moral feeling; of warm attachment and steady friendship. The intense interest displayed by the public, in Edinburgh, during the progress of his illness, and the profound and universal sorrow expressed on the tidings of his death, significantly testify the estimation in which he was so deservedly held.

